# Correction: Novel ^90^Sr analysis of environmental samples by Ion-Laser InterAction Mass Spectrometry

**DOI:** 10.1039/d2ay90110b

**Published:** 2022-08-23

**Authors:** Maki Honda, Martin Martschini, Oscar Marchhart, Alfred Priller, Peter Steier, Robin Golser, Tetsuya K. Sato, Tsukada Kazuaki, Aya Sakaguchi

**Affiliations:** Faculty of Physics, Isotope Physics, University of Vienna Währinger Strasse 17 Vienna 1090 Austria honda.maki@jaea.go.jp; Japan Atomic Energy Agency 2-4 Shirakata, Tokai Ibaraki 319-1195 Japan; Center for Research in Isotopes and Environmental Dynamics, University of Tsukuba 1-1-1 Tennodai, Tsukuba Ibaraki 305-8577 Japan

## Abstract

Correction for ‘Novel ^90^Sr analysis of environmental samples by Ion-Laser InterAction Mass Spectrometry’ by Maki Honda *et al.*, *Anal. Methods*, 2022, **14**, 2732–2738, https://doi.org/10.1039/D2AY00604A.

In the original article, in the sentence in the introduction beginning “Previous studies have reported LODs of <1 Bq…”, the number of atoms approximately equal to 1 Bq was incorrect. The corrected sentence is given below.

Previous studies have reported LODs of <1 Bq (1 Bq ≈ 1.3 × 10^9^ atoms) by ICP-MS/MS,^8,9^ 4 mBq by RIMS,^11^ and <1 mBq by TIMS.^12,13^

The Royal Society of Chemistry apologises for these errors and any consequent inconvenience to authors and readers.

## Supplementary Material

